# Long-term follow-up of thalamic deep brain stimulation for essential tremor – patient satisfaction and mortality

**DOI:** 10.1186/1471-2377-14-120

**Published:** 2014-06-05

**Authors:** Mari Naumann Børretzen, Silje Bjerknes, Terje Sæhle, Mona Skjelland, Inger Marie Skogseid, Mathias Toft, Espen Dietrichs

**Affiliations:** 1 Department of Neurology, Oslo University Hospital, PO Box 4950, Nydalen N-0424 Oslo, Norway; 2 Faculty of Medicine, University of Oslo, Oslo, Norway; 3 Department of Neurosurgery, Oslo University Hospital, Oslo, Norway

**Keywords:** Essential tremor, Deep brain stimulation, Mortality

## Abstract

**Background:**

Ventral intermediate thalamic nucleus (VIM) deep brain stimulation (DBS) is an effective treatment for tremor, but there is limited data on long-term efficacy and mortality after VIM-DBS. Here we report the analysis of patient satisfaction and mortality in all patients treated in our center 1996–2010 with VIM-DBS for essential tremor (ET).

**Methods:**

Forty-six consecutive patients were included in this study. Medical records were reviewed, and a follow-up questionnaire was sent to all surviving patients.

**Results:**

Seventy percent of all possible participants (26 patients) answered the questionnaire. Follow-up time for the responding patients was median 6.0 years (2–16). Median self-reported score on visual analogue scale of the initial postoperative effect on tremor was 8.5 (0.1–10), with a significant reduction to 7.4 (0–10) at follow-up (p = 0.001). Patients reported a median score of 10 (0–10) for overall patient satisfaction with VIM-DBS treatment. Eight patients (17%) died after median 8.9 years (0.6–15) after surgery, at median age 77.4 years (70–89). One patient (2%) committed suicide seven months after the operation. Calculated standard mortality ratio among ET patients was 1.3 (CI 0.6–2.6), similar to the general population.

**Conclusion:**

We found no significant increase in mortality in this cohort of VIM-DBS operated ET patients compared to the general population in Norway. The patients reported high long-term satisfaction and continuing effect of VIM-DBS on tremor even after many years. VIM-DBS therefore seems to be an effective symptomatic long-term treatment of ET. However, one patient committed suicide. Only one other suicide has previously been reported after VIM-DBS. It is therefore still unclear whether VIM-DBS increases suicide risk.

## Background

Tremor is a common sign in movement disorders and can in advanced cases lead to severe loss of daily function. Deep brain stimulation (DBS) of the ventral intermediate nucleus (VIM) of the thalamus was introduced by Benabid et al. [1,2] for tremor in Parkinson’s disease (PD) in 1987 and for essential tremor (ET) in 1991. Today DBS is commonly used in PD, but the subthalamic nucleus is usually the preferred target since VIM-DBS is not efficient for other parkinsonian symptoms [3]. VIM-DBS can also be valuable in dystonic tremor [4], but the internal segment of pallidum is usually the preferred target in dystonic patients [5].

Several studies have confirmed that VIM-DBS is effective in ET [6-9], and the method is well established as a symptomatic treatment for severe medically resistant tremor. There are however, several reports of a gradually diminishing effect on tremor over time [10,11]. To our knowledge there are no previous studies of mortality after DBS for ET. Some authors describe an increased rate of suicide following DBS. Most of these articles address the incidence of suicide after STN-DBS [12]. We have only found one previous description of a suicide after VIM-DBS [13].

We have performed a retrospective study of all patients with ET receiving VIM-DBS in our hospital from 1996 to 2010. Herein we report analysis of patient satisfaction and self-reported effect of VIM-DBS, adverse effects and mortality.

## Methods

### Study population and data acquisition

All 46 patients who underwent VIM-DBS surgery for ET at Oslo University Hospital from 1996 to 2010 were included in this retrospective study. The indication for VIM-DBS was severe and incapacitating tremor with unsatisfactory response to medical treatment. ET was diagnosed according to the consensus statement of the Movement Disorder Society on tremor [14]. All patients were followed until November 1, 2012, or death.

Data were obtained from patient records, from a new 20-item questionnaire (in Norwegian; Additional files 1 and 2) sent to all surviving patients, and from the Norwegian Death Registry. Death statistics were obtained from Statistics Norway (SSB), and mortality data for one year (2006) representative for the period were used. In the 20-item questionnaire, overall patient satisfaction and self-reported treatment effect were measured by visual analog scales (VAS). The patients were asked to report treatment effect both during the initial postoperative period after a stable effect of VIM-DBS had been obtained, and after long-term treatment (in 2012). The questionnaire also contained questions about work performance and psychiatric co-morbidity. Written informed consent was obtained from all participants. The Regional Committee for Medical and Health Research Ethics in South-East Norway approved the study (REK 2011/2459), and permissions to use data from deceased patients were obtained from The Norwegian Directorate of Health and The Norwegian Data Protection Authority.

### Surgical procedure

Preoperative MRI sequences from the day before the operation were merged with a stereotactic CT scan performed after the stereotactic frame had been mounted. Targets and electrode trajectory were planned using the iPlan®(version 3.0) computer-aided neuronavigation system (BrainLAB, München, Germany). The VIM target was planned using our standard stereotactic coordinates in relation to the anterior commissure (AC)-posterior commissure (PC) line: approximately 30% of the AC-PC distance anterior to PC, 50% of the AC-PC distance lateral of the midline and 0-2 mm superior to the AC-PC line. The target was adjusted in some patients with abnormal ventricles and according to the symptoms, so that the target was 12 mm (narrow ventricles and main symptoms in lower extremity) - 16 mm (wide ventricles and main symptoms in upper extremity) lateral to the midline. After clinical test stimulation in the awake patient, confirming good tremor suppression and no unacceptable side effects, a permanent quadripolar electrode (model 3387 or 3389, Medtronic, MN, USA) was inserted. Electrode position was checked using perioperative radiography. With six exceptions all patients were operated bilaterally. Both electrodes were implanted during the same operation. Finally, the electrodes were connected to a subcutaneous infraclavicular pulsegenerator (Kinetra, Soletra and Activa PC from Medtronic) via a subcutaneous extension cable. This was done under general anesthesia. Of the 40 patients receiving bilateral stimulation, two had previously received unilateral stimulation. Four patients had earlier been treated with unilateral thalamotomy.

### Statistical analysis

Many of the measured parameters were not normally distributed, and non-parametric tests were used in the analysis. P-value < 0.05 was considered statistically significant. Mortality in ET patients was compared to the general population of Norway using standard mortality ratio (SMR). SPSS software version 16.0. was used for statistical analysis.

## Results

### Outcome

There were 14 (30%) women and 32 (70%) men included in the study. Median follow-up time was 5.5 years (0.6-16) and median age at final evaluation was 71.6 years (39–90).

Among all 46 patients, eight had died and one was unable to participate due to cognitive impairment. Thus, 37 patients received the questionnaire, and twenty-six of these (70%) responded. Median follow-up time of the responding patients was 6.0 years (2–16).

Stimulation parameters registered in the patient journal 3–8 years after surgery were available in 30 patients (Table 1B). A significant increase was seen from the first postoperative year to follow-up, both in median voltage, 2.3 V to 3.5 V (p = 0.001), and frequency, 148 Hz to 181 Hz (p = 0.001). The median pulse width was unchanged.

**Table 1:** Data from patient records

**A: Epidemiological characteristics of patients treated with VIM-DBS**	
	All (N = 46)
Sex M/F	32/14
Age at operation	65.2 (29.5-84.2)
Earlier unilateral DBS	2
Earlier thalamotomi	4
**B: Stimulation parameters during long-term VIM-DBS treatment**	
Unilateral/bilateral	4/26
Voltage postoperative	2.3 (1.5–4)
Voltage follow-up	3.5 (2.1–4.4)
Puls width postoperative	90 (60–120)
Puls width follow-up	90 (60–120)
Frequency postoperative	148 (90–230)
Frequency follow-up	181 (120–230)

Data from patient records presented as median (range). Follow-up until 01.11.12 or death 1A. M: male, F: female. 1B. All: essential tremor patients.

Effect of VIM-DBS on tremor was reported retrospectively in the questionnaire by 26 patients (Table 2B). Median self-reported VAS-score on initial postoperative effect was 8.5 (0.1-10) in all responders. This was significantly reduced to 7.4 (0.1-10) at follow-up (p = 0.001). The median score for overall satisfaction with VIM-DBS treatment was 10 (0–10).

**Table 2:** Data from patient self-report questionnaires

**A: Medication for tremor**			
Number of patients N = 26	Yes	No	Unknown
Preoperative	12 (46)	11 (42)	3 (12)
Postoperative	5 (19)	16 (62)	5 (19)
At follow-up	9 (35)	12 (46)	5 (19)
**B: Patient self-report**			
	All (N = 26)		
VIM-DBS effect on tremor postoperative	8.5 (0.1-10)		
VIM-DBS effect on tremor at follow-up	7.4 (0.1-10)		
Overall satisfaction with VIM-DBS	10 (0–10)		
**C: Most common adverse effects with VIM-DBS**			
Variable	All		
Number of patients	26		
Dysarthria	17 (65)		
Headache	9 (35)		
Parestesia	6 (23)		
Abnormal taste	8 (31)		
Dizziness	5 (19)		
Discomfort tongue	4 (15)		
Reduced balance or coordination	4 (15)		
Other	8 (31)		

Patient self-report from follow-up questionnaire. Median follow-up 6.0 years (range, 2–16).

2A. Data are given in number of patients = n (%) reporting use of medication for tremor including betablockers, antiepileptics, benzodiazepines.

2B. VAS scale 1–10, where 0 is no effect of VIM-DBS on tremor and 10 is maximum effect.

2C. Data given in number of patients = n (%) experiencing adverse effects.

VIM-DBS showed no statistically significant effect on the working situation in our patients. Eight of 26 responding patients reported to be on disability benefit before VIM-DBS surgery. Only one patient reported to regain full working ability after VIM-DBS treatment, while one patient reported to be able to work part time.

### Adverse effects and battery replacements

Serious adverse effects were rare. One patient (2%) developed a superficial infection nine days after implantation and was treated with antibiotics. Table 2C shows self-reported adverse effects after VIM-DBS. There was no significant increase in self-reported psychiatric symptoms after VIM-DBS implantation. However, one patient reported a temporary increase of depressive symptoms after surgery that had declined until follow-up. Another patient with a previous history of depression got an exacerbation of depressive symptoms, that did not decline until follow-up.

On November 1, 2012 the original battery was still functioning in 14 patients after median 2.7 years (2–6.3). The battery had been replaced in 29 patients after median 3.7 years (2.0-8.5). In connection with battery replacement, three (10%) patients got an infection and were treated with antibiotics. One of these developed meningitis and the electrodes were removed, but were later replaced.

### Mortality

**Figure 1:**
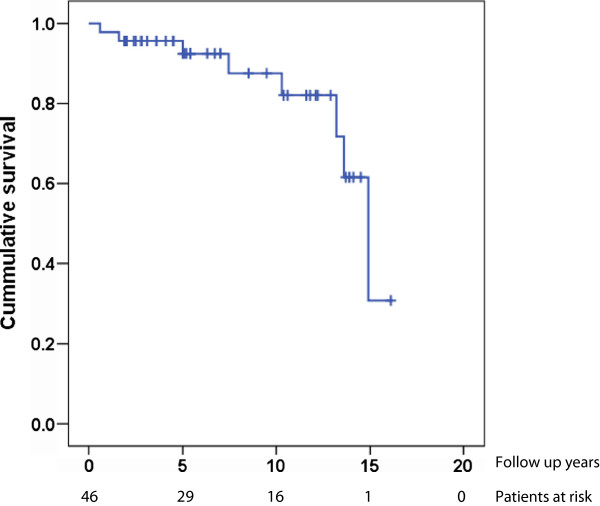
Kaplan Meier curve constructed using death as outcome.

Eight patients (17%) died during the follow-up period (2 women, 6 men). The median time from surgery to death was 8.9 years (0.6-15), and the median age at death was 77.4 years (70–89). Median age at surgery among the patients who died during follow-up was 71.8 (60–84), compared to a median age at surgery of 65.2 for those who survived. One patient (2%) committed suicide during an episode of acute depression about seven months after surgery. This 73 year old ET patient had to our knowledge no prior history of psychiatric disease, and DBS tremor suppression was excellent at the last examination six months after VIM-DBS surgery.The mortality in ET patients was calculated using standard mortality ratio (SMR). Calculated expected deaths were 6.35, while eight deaths were observed. This gave a standard mortality ratio of 1.26 with a 95% confidence interval of 0.54 - 2.48 (p > 0.05). 10 years survival rate after VIM-DBS operation was above 80% (Figure 1).

## Discussion

Our study shows that the total survival 10 years after VIM-DBS implantation in ET patients (Figure 1) is above 80%. Louis et al. [15] have indicated that the mortality in ET patients not treated with DBS is increased relative to a control group without ET. We found no significant increase in mortality in VIM-DBS treated ET patients compared to the general Norwegian population (calculated SMR 1.26).

There are several limitations to this comparison of mortality in ET patients. The total number of patients included in this study is relatively small. Because of this, a possible modest increase in mortality would not give a significant increase in SMR and could therefore escape recognition. However, the group of patients included is relatively large compared to most other studies of ET patients. Another factor that might affect our results could be that ET patients selected for surgery have less comorbidity than those excluded.

One of our VIM-DBS patients committed suicide within seven months after surgery. Increased suicide rate after DBS has been indicated by several authors, but we have found only one previous description of a suicide after VIM-DBS [16]. Burkhard et al. [13] described a 4.3% suicide rate after DBS, and found no relationship to the underlying condition or DBS target. Most reports have discussed suicide risk after subthalamic nucleus DBS in PD patients [12], but a recent randomized, controlled multicentre study found no direct association between DBS surgery and an increased risk for suicide ideation and behaviors in PD patients [16]. Therefore, it is still unclear whether, or to which extent, there is an increased suicide risk after DBS, and whether the risk varies between different DBS targets.

The results from the patient questionnaire are hampered by its retrospective design, with both recall- and selection bias. Another problem is the wide range of follow-up times due to the low number of patients operated each year. Patient satisfaction is difficult to assess, and there are no validated methods to measure this in our patient group. We used VAS-scores, since these have shown to have good validity and reliability for patient satisfaction after other interventions [17]. Seventy percent of the eligible participants responded to the questionnaire. The median VAS-score for the postoperative effect of VIM-DBS on tremor reported was high (Table 2B). At follow-up, a modest, but significant effect reduction was reported compared to the postoperative score. In spite of this, a median VAS-score of 7.4 for the effect of VIM-DBS on tremor after median 6.0 years follow-up, indicate that VIM-DBS has a good long-term effect on ET in most patients (Table 2A). Median VAS-score of overall patient satisfaction with VIM-DBS treatment in the questionnaire responders was high.

VAS-scale was also used by Zhang et al. [18] in their long term study. At 56.9 months follow-up they found a 1.43 (±2.62) mean score for the effect of VIM-DBS on tremor in ET patients. Some other studies have also reported a decrease in activities of daily living (ADL) and an increase in tremor at long-term follow-up, indicating a loss of benefit of VIM-DBS over time or disease progression [10,11]. Compared to these reports, our results seem to indicate a better long-term effect of DBS on tremor, similar to what was described in a few other long-term studies of ET [6,7,9].

Stimulation parameters were increased both for voltage and frequency from the first postoperative year until follow-up. These findings are similar to previous reports on ET [6,7,9]. Whether this increase in stimulation parameters and the loss of effect of VIM-DBS on tremor in ET patients is related to disease progression or tolerance to the VIM-DBS treatment, is still unclear.

Dysarthria was the most frequent reported adverse effect. Similar findings have been reported from other DBS studies, especially after bilateral stimulation [9,19]. One of two responding patients with unilateral VIM-DBS also reported dysarthria in the follow up questionnaire. Disequilibrium and balance difficulty together with reported falls have also been used as a caution against bilateral stimulation [9]. However, coordination problems and dizziness were not reported as severe problems in our study (Table 2C).

As in other studies of patients with ET [9,10] there is a larger proportion of men compared to women treated with DBS (2/3), despite that the prevalence of ET seems to be equal in men and women [20]. Whether this has a cultural or a gender dependent explanation is difficult to tell, but similar is observed also in PD patients treated with DBS [21-23].

## Conclusions

We found no significant increase in mortality in ET patients treated with VIM-DBS when compared to the general population of Norway. The estimated overall survival rate after VIM-DBS is high, but one patient committed suicide seven months after surgery. The adverse effects are generally well tolerated, and the patients report high long-term satisfaction with the treatment, with a continuing effect of VIM-DBS on tremor. Bilateral VIM-DBS therefore seems to be an effective symptomatic long-term treatment of ET, with a favorable risk-benefit profile.

## Competing interests

Financial disclosure: Silje Bjerknes and Terje Sæhle have received travel grants from Medtronic. Inger Marie Skogseid has received honoraria for lectures and financial support to attend meetings arranged by Medtronic, and has participated in studies of DBS in dystonia that have received financial support from Medtronic. Mathias Toft has received honoraria for lecturing from Medtronic, and research grants from the Research Council of Norway and the South-Eastern Norway Regional Health Authority. Espen Dietrichs has received honoraria for lecturing and traveling grants from Medtronic, and research grants from the South-Eastern Norway Regional Health Authority. Mari Naumann Børretzen and Mona Skjelland have nothing to declare.

## Authors’ contributions

MNB participated in the design of the study, collected and analyzed the information from the questionnaires and patient files, and drafted the manuscript. SB, TS, MS and MS all participated in the analysis of the results and helped to draft the manuscript. MT participated in the design of the study, in the analysis of the results, and helped to draft the manuscript. ED conceived the study, and participated in its design and coordination and helped to draft the manuscript. All authors read and approved the final manuscript.

## Pre-publication history

The pre-publication history for this paper can be accessed here:

http://www.biomedcentral.com/1471-2377/14/120/prepub

## Supplementary Material

Additional file 1 Patient self-report questionnaire (Norwegian original). Click here for file

Additional file 2 Patient self-report questionnaire (English translation). Click here for file

## References

[B1] Benabid AL Pollak P Louveau A Henry S de Rougemont J Combined (thalamotomy and stimulation) stereotactic surgery of the VIM thalamic nucleus for bilateral Parkinson disease Appl Neurophysiol 1987 50 344 346 3329873 10.1159/000100803

[B2] Benabid AL Pollak P Gervason C Hoffmann D Gao DM Hommel M Perret JE de Rougemont J Long-term suppression of tremor by chronic stimulation of the ventral intermediate thalamic nucleus Lancet 1991 337 403 406 10.1016/0140-6736(91)91175-T 1671433

[B3] Ferreira JJ Katzenschlager R Bloem BR Bonuccelli U Burn D Deuschl G Dietrichs E Fabbrini G Friedman A Kanovsky P Kostic V Nieuwboer A Odin P Poewe W Rascol O Sampaio C Schüpbach M Tolosa E Trenkwalder C Schapira A Berardelli A Oertel WH Summary of the recommendations of the EFNS/MDS-ES review on therapeutic management of Parkinson’s disease Eur J Neurol 2013 20 5 15 10.1111/j.1468-1331.2012.03866.x 23279439

[B4] Morishita T Foote KD Haq IU Zeilman P Jacobson CE Okun MS Should we consider Vim thalamic deep brain stimulation for select cases of severe refractory dystonic tremor Stereotact Funct Neurosurg 2010 88 98 104 10.1159/000289354 20197711

[B5] Volkmann J Wolters A Kupsch A Müller J Kühn AA Schneider GH Poewe W Hering S Eisner W Müller JU Deuschl G Pinsker MO Skogseid IM Roeste GK Krause M Tronnier V Schnitzler A Voges J Nikkhah G Vesper J Classen J Naumann M Benecke R DBS study group for dystonia. Pallidal deep brain stimulation in patients with primary generalised or segmental dystonia: 5-year follow-up of a randomised trial Lancet Neurol 2012 11 1029 1038 10.1016/S1474-4422(12)70257-0 23123071

[B6] Rehncrona S Johnels B Widner H Tornqvist AL Hariz M Sydow O Long-term eficacy of thalamic deep brain stimulation for tremor: double-blind assessments Mov Disord 2003 18 163 170 10.1002/mds.10309 12539209

[B7] Sydow O Thobois S Alesch F Speelman JD Multicentre European study of thalamic stimulation in essential tremor: a six year follow up J Neurol Neurosurg Psychiatry 2003 74 1387 1391 10.1136/jnnp.74.10.1387 14570831 PMC1757400

[B8] Pahwa R Lyons KL Wilkinson SB Carpenter MA Tröster AI Searl JP Overman J Pickering S Koller WC Bilateral thalamic stimulation for the treatment of essential tremor Neurology 1999 53 1447 1450 10.1212/WNL.53.7.1447 10534249

[B9] Pahwa R Lyons KE Wilkinson SB Simpson RK Jr Ondo WG Tarsy D Norregaard T Hubble JP Smith DA Hauser RA Jankovic J Long-term evaluation of deep brain stimulation of the thalamus J Neurosurg 2006 104 506 512 10.3171/jns.2006.104.4.506 16619653

[B10] Hariz GM Blomstedt P Koskinen LO Long term effect for deep brain stimulation for essential tremor on activities of daily living and health related quality of life Acta Neurol Scand 2008 118 387 394 10.1111/j.1600-0404.2008.01065.x 18616684

[B11] Kumar R Lozano AM Sime E Lang AE Long-term follow up of thalamic deep brain stimulation for essential and parkinsonian tremor Neurology 2003 61 1601 1604 10.1212/01.WNL.0000096012.07360.1C 14663050

[B12] Voon V Krack P Lang AE Lozano AM Dujardin K Schüpbach M D’Ambrosia J Thobois S Tamma F Herzog J Speelman JD Samanta J Kubu C Rossignol H Poon YY Saint-Cyr JA Ardouin C Moro E A multicentre study on suicide outcomes following subthalamic stimulation for Parkinson’s disease Brain 2008 131 2720 2728 10.1093/brain/awn214 18941146 PMC2724899

[B13] Burkhard PR Vingerhoets FJ Berney A Bogousslavsky J Villemure JG Ghika J Suicide after successful deep brain stimulation for movement disorders Neurology 2004 63 2170 2172 10.1212/01.WNL.0000145603.48221.B5 15596774

[B14] Deuschl G Bain P Brin M Consensus statement of the movement disorder society on tremor Ad Hoc Scientific Committee Mov Disord 1998 13 2 23 10.1002/mds.870131303 9827589

[B15] Louis ED Benito-León J Ottman R Bermejo-Pareja F A population based study of mortality in essential tremor Neurology 2007 96 1982 1989 18025392 10.1212/01.wnl.0000279339.87987.d7

[B16] Weintraub D Duda JE Carlson K Luo P Sagher O Stern M Follett KA Reda D Weaver FM Suicide ideation and behaviours after STN and GPi DBS surgery for Parkinson’s disease: results from a randomised, controlled trial J Neurol Neurosurg Psychiatry 2013 84 1113 1118 10.1136/jnnp-2012-304396 23667214 PMC4594869

[B17] Brokelman RB Haverkamp D van Loon C Hol A van Kampen A Veth R The validation of the visual analogue scale for patient satisfaction after total hip arthroplasty Eur Orthop Traumatol 2012 3 101 105 10.1007/s12570-012-0100-3 22798966 PMC3389603

[B18] Zhang K Bhatia S Oh MY Cohen D Angle C Whiting D Long term results of thalamic deep brain stimulation for essential tremor J Neurosurg 2010 112 1271 1276 10.3171/2009.10.JNS09371 19911883

[B19] Putzke JD Wharen RE Jr Obwegeser AA Wszolek ZK Lucas JA Turk MF Uitti RJ Thalamic deep brain stimulation for essential tremor: recommendations for long- term outcome analysis Can J Neurol Sci 2004 31 333 342 15376477 10.1017/s0317167100003413

[B20] Lou JS Jankovic J Essential tremor: clinical correlates in 350 patients Neurology 1991 41 234 238 10.1212/WNL.41.2_Part_1.234 1992367

[B21] Hariz G Hariz MI Gender distribution in surgery for Parkinson’s disease Parkinsonism Relat Disord 2000 6 155 157 10.1016/S1353-8020(00)00009-2 10817954

[B22] Hariz GM Nakajima T Limousin P Foltynie T Zrinzo L Jahanshahi M Hamberg K Gender distribution of patients with Parkinson’s disease treated with subthalamic deep brain stimulation; a review of the 2000–2009 literature Parkinsonism Relat Disord 2011 17 146 149 10.1016/j.parkreldis.2010.12.002 21195012

[B23] Eskandar EN Flaherty A Cosgrove GR Shinobu LA Barker FG II Surgery for Parkinson disease in the United States, 1996 to 2000: practice patterns, short-term outcomes, and hospital charges in a nationwide sample J Neurosurg 2003 99 863 871 10.3171/jns.2003.99.5.0863 14609166

