# Supplementary material for: Long-term follow-up of thalamic deep brain stimulation for essential tremor – patient satisfaction and mortality
Source: BMC Neurol. 2014 Jun 5;14:120. doi: 10.1186/1471-2377-14-120 (PMC4052337; doi:10.1186/1471-2377-14-120)
Supplement: Additional file 1 — Patient self-report questionnaire (Norwegian original). [file 1471-2377-14-120-S1.pdf]

**SPØRRESKJEMA FOR PASIENTER MED DYP HJERNESTIMULERING**

Kontroller at data stemmer og sett kryss eller fyll inn:

| | |
|---|---|
| **NAVN:** | |
| **FØDT:** | **OPERASJONSDATO:** |

**1. YRKESSTATUS <u>FØR</u> 1. GANGS OPERASJON:**

Yrke:

| | | | | |
|---|---|---|---|---|
| ☐ I full jobb | ☐ Ikke i fast arbeid | ☐ Delvis sykemeldt | ☐ ufør | ☐ alderspensjonert |

**2. YRKESSTATUS <u>RETT ETTER</u> 1.GANGS OPERASJON**

| | | | | |
|---|---|---|---|---|
| ☐ I full jobb | ☐ Ikke i fast arbeid | ☐ delvis sykemeldt | ☐ ufør | ☐ alderspensjonert |

**3. YRKESSTATUS <u>IDAG</u>:**

Samme yrke som før operasjon:

| | |
|---|---|
| ☐ Ja | ☐ Nei<br>Nytt yrke: |

| | | | | |
|---|---|---|---|---|
| ☐ I full jobb | ☐ Ikke i fast arbeid | ☐ delvis sykemeldt | ☐ ufør | ☐ alderspensjonert |

**4.MEDIKAMENTBRUK FØR 1. GANGS OPERASJON**

| | |
|---|---|
| ☐ Nei: | ☐ Ja<br>Angi medikament/-er: |

**5.MEDIKAMENTBRUK RETT ETTER 1. GANGS OPERASJON**

| | |
|---|---|
| ☐ Nei: | ☐ Ja<br>Angi medikament/-er |

| 6. MEDIKAMETBRUK IDAG: | |
|---|---|
| ▢ Nei | ▢ Ja<br>Angi medikament/-er: |

| 7. ANDRE/ TIDLIGERE SYKDOMMER | | | |
|---|---|---|---|
| ▢ epilepsi | ▢ Angst<br>▢ depresjon | ▢ lungekreft | ▢ Annen kreft:<br>Type: |
| ▢ astma | ▢ Dupuytrens kontraktur (sammentrekning av sener i håndflaten) | ▢ Grønn stær | ▢ Grå stær |
| Annet: | | ▢ Født for tidlig<br>- Hvor mye for tidlig: | |

**8. LED DU AV DEPRESJON FØR OPERASJONEN**

Sett strek på linjen for hva som passer for deg for hvor deprimert du var før operasjonen. Der linjen går fra ikke deprimert til dypt deprimert.

Ikke deprimert ———————————————————— Dypt deprimert

**9.VAR DU DEPRIMERT RETT ETTER OPERASJON**

Sett strek på linjen for hva som passer for deg for hvor deprimert du var før operasjonen.
Der linjen går fra ikke deprimert til dypt deprimert.

Ikke deprimert ———————————————————— Dypt deprimert

**10. ER DU DEPRIMERT IDAG**

Sett strek på linjen for hva som passer for deg for hvor deprimert du var før operasjonen.
Der linjen går fra ikke deprimert til dypt deprimert.

Ikke deprimert ________________________________ Dypt deprimert

**11. LED DU AV ANGST FØR OPERASJONEN**

Sett strek på linjen for hva som passer for deg for hvor mye du led av angst før operasjonen. Der linjen går fra ingen angst plager til invalidiserende angst.

Ingen angst ________________________________ invalidiserende angst

**12.LED DU AV ANGST RETT ETTER OPERASJONEN**

Sett strek på linjen for hva som passer for deg for hvor mye du led av angst etter operasjonen.
Linjen går fra ingen angst plager til invalidiserende angst.

Ingen angst ________________________________ invalidiserende angst

**13. LIDER DU AV ANGST IDAG**

Sett strek på linjen for hva som passer for deg for hvor mye du led av angst før operasjonen.

Ingen angst ________________________________ invalidiserende angst

**14. BIVIRKINGER ETTER DYP HJERESTIMULERING**

| | | |
|---|---|---|
| ☐ Nummenhet/ prikking | ☐ hodepine | ☐ smaksforandring |
| ☐ taleproblemer | ☐ Ubehag på tungespiss | ☐ svimmelhet |

☐ Annet:

**15. OPPLEVD EFFEKT AV DYP HJERNESTIMULATOR RETT ETTER 1.GANGS OPERASJON**

Sett en strek på linja  etter hva som passer for deg fra ingen opplevd effekt til at alle plager ble borte.

Ingen effekt ______________________________ alle plager borte

**16. OPPLEVD EFFEKT AV DBS STIMULATOR IDAG**

Sett en strek på linja  etter hva som passer for deg fra ingen opplevd effekt til at alle plager ble borte.

Ingen effekt ______________________________ alle plager borte

**17. HVIS EFFEKT HAR BLITT DÅRLIGERE:**
**HVOR LENGE HADDE DU GOD EFFEKT AV OPERASJONEN?**

Angi antall år: ________

**18. HVOR TILFREDS ER DU IDAG  MED Å HA GJENNOMFØRT DYP HJERNESTIMULERING**

Sett en strek på linja etter hvor tilfreds du er med å ha gjennomført dyp hjernestimulering der linja går fra svært misfornøyd til svært fornøyd.

Svært misfornornøyd ______________________________ svært fornøyd

**EVENTUELLE KOMMENTARER TIL SPØRSMÅL 18**

**GENERELLE KOMMENTARER:**
