# Supplementary material for: Long-term follow-up of thalamic deep brain stimulation for essential tremor – patient satisfaction and mortality
Source: BMC Neurol. 2014 Jun 5;14:120. doi: 10.1186/1471-2377-14-120 (PMC4052337; doi:10.1186/1471-2377-14-120)
Supplement: Additional file 2 — Patient self-report questionnaire (English translation). [file 1471-2377-14-120-S2.pdf]

Deep brain stimulation for essential tremor

Follow-up questionnaire for patients

Personal data:
Name:
Date of birth:
Date of operation for deep brain stimulation:

Please tick all relevant boxes, if needed please give further details below

1. **Status of work situation before operation:**
    a. Full time job ☐
    b. No work ☐
    c. Partly sick leave ☐
    d. Disability benefit ☐
    e. Retired ☐

2. **Status of work situation after operation**
    a. Full time job ☐
    b. No work ☐
    c. Partly sick leave ☐
    d. Disability benefit ☐
    e. Retired ☐

3. **Status of work situation today:**
    a. Same profession as before operation ☐
    b. New profession after the operation ☐
    Describe: ____

        i. Full time job ☐
        ii. No work ☐
        iii. Partly sick leave ☐
        iv. Disability benefit ☐
        v. Retired ☐

4. **Any use of medication before the operation:**
    a. No ☐
    b. Yes ☐
    Describe what type of medication you used:
    ____

5. **Any use of medication after the operation:**
   a. No ☐
   b. Yes ☐
   Describe what kind of medication you used:
   ____

6. **Any use of medication today:**
   a. No ☐
   b. Yes ☐
   Describe what kind of medication you use:
   ____

7. **Other diseases:**
   a. Epilepsy ☐
   b. Anxiety ☐
   c. Depression ☐
   d. Lung-cancer ☐
   e. Other cancer ☐ Type: ______________________
   f. Asthma ☐
   g. Dupuytrens contraction ☐
   (fixed contraction of the hand)
   h. Glaucoma ☐
   i. Cataract ☐
   j. Heart disease ☐ Type: ______________________
   k. Other ☐ Type: ______________________

8. **Were you born before term?**
   a. No ☐
   b. Yes ☐ Number of days:______________

9. **Did you suffer from depression before the operation** (please place a vertical mark on the line to indicate how depressed you were)?

   Not depressed ____ Deeply depressed

10. **Were you depressed after the operation** (please place a vertical mark on the line to indicate how depressed you were)?

    Not depressed ____ Deeply depressed

11. **Do you feel depressed today** (please place a vertical mark on the line to indicate how depressed you are)?

    Not depressed ____ Deeply depressed

12. **Did you suffer from anxiety before the operation** (please place a vertical mark on the line to indicate how much you suffered from anxiety)?

No anxiety ______________________________ Incapacitating anxiety

13. **Did you suffer from anxiety after the operation** (please place a vertical mark on the line to indicate how much you suffered from anxiety)?

No anxiety ______________________________ Incapacitating anxiety

14. **Do you suffer from anxiety today** (please place a vertical mark on the line to indicate how much you suffer from anxiety)?

No anxiety ______________________________ Incapacitating anxiety

15. **Side- effects of deep brain stimulation**
    a. Numbness ☐
    b. Headache ☐
    c. Abnormal taste ☐
    d. Dysarthria ☐
    e. Discomfort tongue ☐
    f. Dizziness ☐
    g. Other ☐
    Type: ______________________________

16. **Self-reported effect of deep brain stimulation on tremor initially after first operation** (please place a vertical mark on the line to indicate the effect from deep brain stimulation on your tremor)?

No effect ______________________________ All tremor gone

17. **Self-reported effect of deep brain stimulation on tremor today** (please place a vertical mark on the line to indicate the effect from deep brain stimulation on your tremor)?

No effect ______________________________ All tremor gone

18. **If you have experienced reduced effect of deep brain stimulation on tremor, how long has this lasted?**
    Number of years or months: ________________

19. Are you satisfied that you have received deep brain stimulation (please place a vertical mark on the line to indicate how satisfied you are)?

Very unsatisfied	______________________________	Very satisfied

Comments:

20. General comments:
